# Body Image Distortion and Exposure to Extreme Body Types: Contingent Adaptation and Cross Adaptation for Self and Other

**DOI:** 10.3389/fnins.2016.00334

**Published:** 2016-07-15

**Authors:** Kevin R. Brooks, Jonathan M. Mond, Richard J. Stevenson, Ian D. Stephen

**Affiliations:** ^1^Department of Psychology, Macquarie UniversitySydney, NSW, Australia; ^2^Faculty of Human Sciences, Perception in Action Research Centre, Macquarie UniversitySydney, NSW, Australia; ^3^Department of Psychology, Centre for Emotional Health, Macquarie UniversitySydney, NSW, Australia; ^4^ARC Centre of Excellence in Cognition and its Disorders, Macquarie UniversitySydney, NSW, Australia

**Keywords:** adaptation, body image, psychophysics, perception, eating disorders, aftereffects, neural representation

## Abstract

Body size misperception is common amongst the general public and is a core component of eating disorders and related conditions. While perennial media exposure to the “thin ideal” has been blamed for this misperception, relatively little research has examined visual adaptation as a potential mechanism. We examined the extent to which the bodies of “self” and “other” are processed by common or separate mechanisms in young women. Using a contingent adaptation paradigm, experiment 1 gave participants prolonged exposure to images both of the self and of another female that had been distorted in opposite directions (e.g., expanded other/contracted self), and assessed the aftereffects using test images both of the self and other. The directions of the resulting perceptual biases were contingent on the test stimulus, establishing at least some separation between the mechanisms encoding these body types. Experiment 2 used a cross adaptation paradigm to further investigate the extent to which these mechanisms are independent. Participants were adapted either to expanded or to contracted images of their own body or that of another female. While adaptation effects were largest when adapting and testing with the same body type, confirming the separation of mechanisms reported in experiment 1, substantial misperceptions were also demonstrated for cross adaptation conditions, demonstrating a degree of overlap in the encoding of self and other. In addition, the evidence of misperception of one's own body following exposure to “thin” and to “fat” others demonstrates the viability of visual adaptation as a model of body image disturbance both for those who underestimate and those who overestimate their own size.

## Introduction

The misperception of one's own body as wider or narrower than its veridical size is a growing health concern, affecting a substantial minority of women and, increasingly, men (Mond et al., 2013; Quick et al., 2014; Griffiths et al., 2016). Those experiencing body size misperception often view themselves as much fatter than they really are. This is thought to be a risk factor for eating-disordered behavior, negative affect, and poor mental health more generally (Stice, 2002; Mond et al., 2013). On the other hand, many overweight and obese individuals misperceive their body as being thinner than it really is (Truesdale and Stevens, 2008; Wetmore and Mokdad, 2012), which has serious implications for physical health (Powell et al., 2010; Yaemsiri et al., 2011).

Along with social pressure to be thin (Stice et al., 2003), body size misperception is often attributed to exposure to unrealistic body ideals as portrayed in the popular media, such as magazines, cinema, and TV. Explanations for the relationship between the media and body image distortions have predominantly focused on sociocognitive processes such as the role of social comparison (Wheeler and Miyake, 1992; Harrison, 2000; Barlett et al., 2008). Such exposure can have negative effects on cognitive schemas concerning one's own body (Stice et al., 1994; Groesz et al., 2001), which is thought to produce the observed changes in body satisfaction (Barlett et al., 2008; Harper and Tiggemann, 2008), perceptions of ideal body size (Stephen and Perera, 2014), and actual judgments of body size and shape itself. Meanwhile, it has been suggested that body size underestimation by larger individuals may result from a form of normalization due to exposure to a population in which obesity is ever more prevalent (Burke et al., 2010; Yaemsiri et al., 2011). However, little is known about the perceptual mechanisms underlying these phenomena.

To students of perception, these observations are reminiscent of a phenomenon known as visual adaptation that has been documented for hundreds or arguably thousands of years (Verstraten, 1996). Adaptation involves prolonged exposure to an “adaptation” stimulus, causing an aftereffect of distorted perception for similar “test” stimuli. Although the original examples involved extended viewing of motion in a particular direction, leading stationary stimuli to appear to drift in the opposite direction (Addams, 1834; Wohlgemuth, 1911), equivalent effects have been documented for other simple stimulus attributes such as orientation (Gibson and Radner, 1937), color (Helmholtz, 1924), depth (Köhler and Emery, 1947), and spatial scale (Blakemore and Sutton, 1969). Such adaptation effects can be brief, resulting from relatively short exposures, or, when the initial exposure is prolonged or when frequent re-exposure is involved, they can be far longer lasting (Webster, 2015).

While these aftereffects provide a degree of amusement, their scientific appeal lies in their ability to reveal the underlying neural mechanisms of perception. The extended period of exposure is known to affect the response properties of neurons in the relevant visual areas of the brain, leading to a temporary imbalance of activity, and a consequent perceptual bias (Barlow and Hill, 1963; Mollon, 1974; Ibbotson, 2005; Krekelberg et al., 2006; Thompson and Burr, 2009). Changes of stimulus attributes can cause observable variations in aftereffect magnitude, allowing us to infer the less easily observable neural processes that underpin these aspects of visual perception, such as the stimulus selectivity of cell populations. For example, by presenting adaptation stimuli in one eye and test stimuli in the other, several researchers have investigated the extent to which stimuli in each eye are processed by common mechanisms (e.g., Wade, 1976). While an absence of aftereffect indicates an independent monocular mechanism for each eye, a substantial “cross adaptation” effect (i.e., evidence of the aftereffect when the adaption and test stimuli appear in different eyes) indicates a binocular processing mechanism. In addition, contingent adaptation effects can provide evidence of a separation of mechanisms for different stimulus types. The original example (McCollough, 1965) involved concurrent adaptation to vertical orange stripes and to horizontal blue stripes, following which vertical test patterns showed a blue-green color aftereffect while horizontal patterns appeared orange. That the aftereffect is contingent on the orientation of the test stimulus suggests that color is processed separately in visual channels that are selective for orientation.

More recently, aftereffects have been demonstrated for more complex stimulus attributes such as the structural properties of a face (Webster and MacLin, 1999; Gwinn and Brooks, 2013). For example, participants who are exposed for a period of time to contracted faces (wherein the features have been digitally altered to be spaced closer together than usual) subsequently see unaltered faces as expanded. Alongside their implications for neural processing, in psychological terms these “high-level” effects are thought to reveal updates to perceptual norms, i.e., changes in the properties of faces that are considered to be average (Rhodes et al., 2005). In addition to face aftereffects, a small number of studies have demonstrated similar phenomena in human bodies. For example, adaptation to a male (female) body (Palumbo et al., 2013) or face (Palumbo et al., 2015) can cause an androgynous body to appear feminine (masculine). Further, participants exposed to images of bodies that have been digitally edited to be thinner than usual subsequently see unaltered bodies as wider than they really are, with reductions of perceived size for those exposed to artificially widened bodies (Winkler and Rhodes, 2005; Glauert et al., 2009; Hummel et al., 2012a,b, 2013; Brooks et al., in press; Mohr et al., 2016). These effects appear to be robust to the specific method of stimulus manipulation, with early studies employing simple changes of aspect ratio (Winkler and Rhodes, 2005), while more recent reports have used synthetic/computer generated images (Glauert et al., 2009), computer-manipulated photographs of human torsos (Hummel et al., 2012a,b, 2013; Mohr et al., 2016), or actual photographs of full human bodies (Robinson and Kirkham, 2014). Further, a recent report has shown independent aftereffects of fat and muscle using morphed stimuli designed to isolate these independent dimensions of body composition (Brooks et al., in press).

While the visual aftereffect paradigm has proven effective for these stimuli, as yet few studies have used this technique to probe the details of the representation of human bodies. In the current study, we present two experiments aimed at probing the extent to which the size of one's own, or of other people's bodies is processed by common, independent, or partially overlapping neural mechanisms. In addition to its theoretical interest in terms of underlying neural structures and perceptual processes, this issue has implications for attempts to account for body image distortion amongst the general public and in clinical populations using visual adaptation processes. For visual adaptation to be considered to be a viable experimental model of body image distortion, some degree of overlap of the representation of self and other would be essential to allow the transfer of aftereffects from media images to the perception of one's own body.

## Experiment 1: Contingent adaptation

The contingent adaptation paradigm has previously been used to infer that the perception of facial structure is mediated by separate neural substrates for faces that differ in terms of race, sex, gender, and even species (Little et al., 2005; Jaquet and Rhodes, 2008; Gwinn and Brooks, 2013, 2015a,b). In the current experiment, we employ the same technique, simultaneously adapting participants to full body images of the self and other that have been manipulated in opposite directions to appear either fatter or thinner than normal. If the perception of body size is mediated by a single neural population for all identities, we should expect both of the opposing adaptation stimuli to affect this mechanism, with their combined effects canceling out. This should result in little or no observable aftereffect for either test stimulus type, and similar results for both conditions. On the other hand, if the processes underlying the perception of body size are to some degree separate for self and other, then the two adaptation stimulus types should affect these separate processes independently.

### Methods

#### Participants

Twenty-four female Caucasian students enrolled in an undergraduate Psychology course at Macquarie University received course credit for their participation. Participants' ages ranged from 18 to 29 years (*M* = 19.5, *SD* = 2.5). All participants had normal or corrected-to-normal vision and gave written informed consent before agreeing to participate. All experiments were conducted in accordance with the declaration of Helsinki and were approved by the Macquarie University Human Research Ethics Committee.

#### Design

The experiment employed a 2 × 2 mixed factorial design, with one within-subjects and one between-subjects independent variable. The within-subjects variable was test identity, with two levels: self and other. The between-subjects variable was adaptation condition with two levels: expanded self/contracted other (Self+/Other−) and contracted self/expanded other (Self−/Other+). The dependent variable was the point of subjective normality (PSN: the stimulus size that appeared normal), which was measured twice—once before (baseline) and once after adaptation. The difference between these two scores (PSN change) was calculated for each individual and the means across participants were subjected to analysis in a factorial ANOVA. Specifically, after adaptation to expanded images of the self and contracted images of the other body, a larger image of the self would be expected to be subjectively normal, whereas a smaller image of others will appear normal. Conversely, for participants adapted to contracted images of the self but expanded images of the other body, the point of subjective normality should be decreased for the self, but increased for others. This pattern of results corresponds to a cross-over interaction in statistical terms.

#### Stimuli

**Figure 1:**
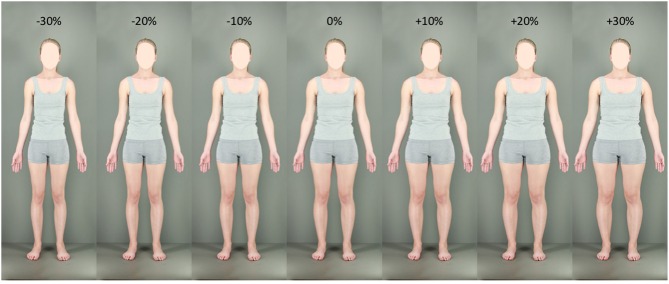
**Examples of test stimuli**. Seven examples, at −30, −20, −10, 0, +10, +20, and +30% size manipulation levels are shown. Although faces have been omitted to preserve subject anonymity, they were fully visible in all parts of each experiment.

Full body images were created from digital photographs of each participant. Participants maintained a neutral expression and posed in standard anatomical position (standing upright, feet positioned approximately at shoulder width, arms straight at the side of the body, hands ~20 cm from the body with palms facing forward, with neutral facial expressions: see Figure 1). All wore standard tight-fitting gray cycling shorts and a gray singlet so that the body shape of each individual was visible. Long hair was pulled back and held in place by a hair band and all jewelry and make-up was removed. Photography took place within a booth painted in Munsell N5 neutral gray located in a room devoid of other lighting. The booth was illuminated with 15 high accuracy d65 fluorescent Philips tubes mounted in high frequency fixtures to reduce the effects of flicker. The camera (Canon EOS 70D) settings (exposure, white balance, ISO, zoom, etc.) were held constant. The photograph displayed the participants' whole body including the head, to ensure recognition of their own images. Weight and height were recorded to obtain a measurement of Body Mass Index (mean BMI = 23.0, *SD* = 4.2). Participants were randomly assigned one unfamiliar “other” body from an existing database of images collected under identical conditions, based on a match of similar BMI and age.

**Figure 2:**
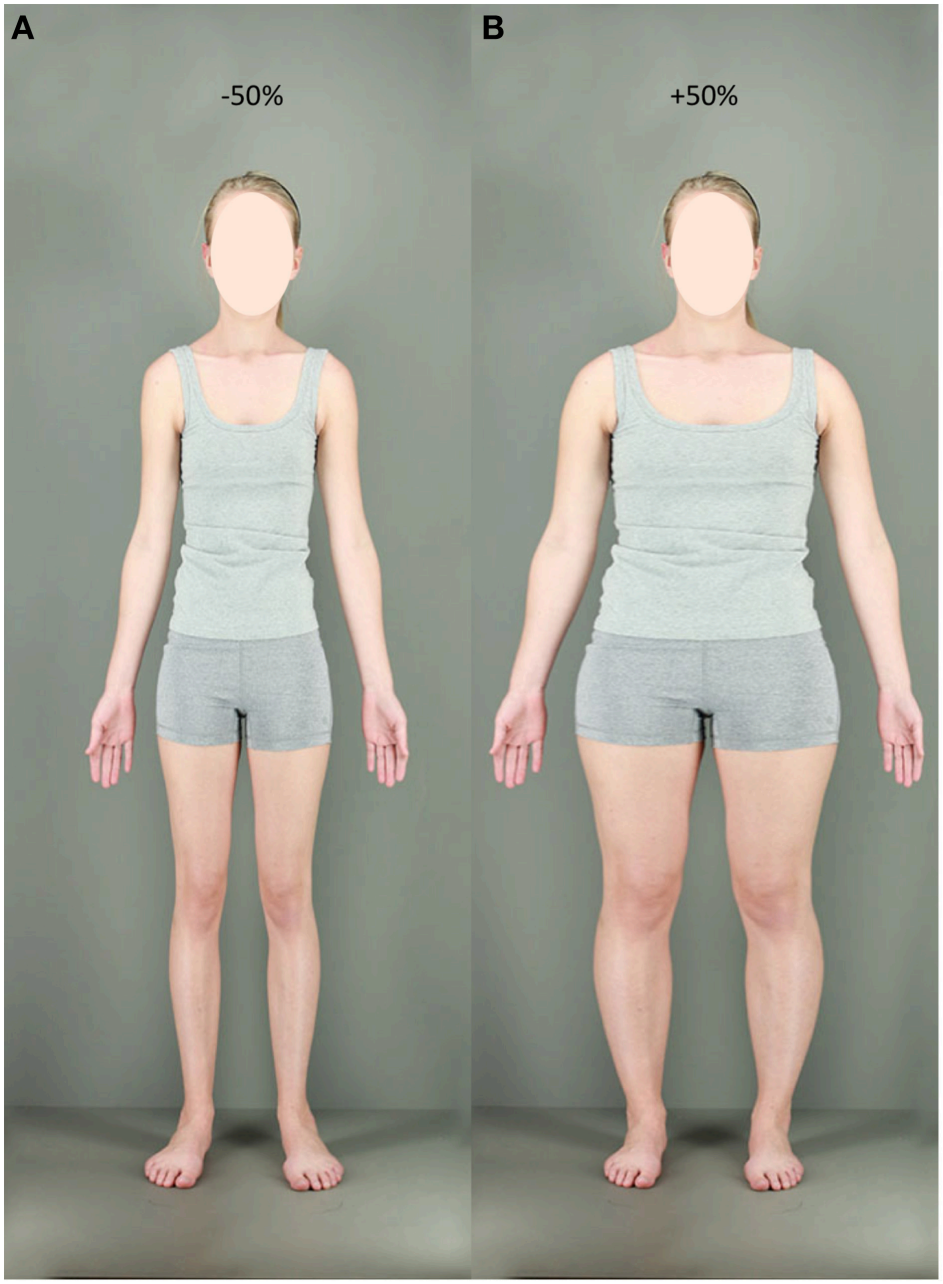
**Examples of adaptation stimuli**. **(A)** Expansion condition at +50%. **(B)** Contraction condition at −50%.

To simulate variations of body size, several versions of each participant's body photograph were created in Adobe Photoshop CS6. Participants' whole bodies from neck to ankles were either horizontally contracted or expanded using the “spherize” function. A feathered marquee allowed these distortions to be blended smoothly into the unmanipulated areas of the image to avoid image discontinuities. Importantly, the size of participants' heads remained un-manipulated to preclude face adaptation effects. Thirteen test images were created, involving spherize manipulations of between −30 and +30% in 5% steps, including the original image (0%). Test images were formatted to a standard height of 720 pixels and a width of 320 pixels. Examples of 7 of these stimuli (omitting −25, −15, −5, +5, +15, and +25% spherize levels due to space constraints) can be seen in Figure 1. Expanded and contracted adaptation images were more extreme, at −50% (Figure 2A) and +50% (Figure 2B) respectively. These were formatted to a standard height of 960 pixels and width of 426 pixels—33% larger than test images—so as to minimize the potential contribution of low-level retinotopic adaptation. All images were presented using Matlab® version 7, operating Psychophysics Toolbox extensions (Kleiner et al., 2007) on a Dell P1130, 21″ color monitor, viewed from a distance of 110 cm.

#### Procedure

The psychophysical task was conducted 2–7 days after stimulus collection. The experiment included image familiarization and a short practice phase followed by a baseline block prior to the adaptation block, and took ~20 min to complete.

Before familiarization, participants were presented with a photograph of the face of a potential “other” partner matched closely for BMI and age, and were asked to indicate whether this person was familiar to them. If participants indicated that they were familiar with the person, another face was shown until an unfamiliar “other” could be established. Participants were then instructed to familiarize themselves with the bodies shown in unmanipulated 13 × 18 cm photographs of the self and their partnered “other” for 1 min. Participants were given an additional min to observe these images between the baseline and adaptation phases.

For baseline data collection, a “yes-no” psychophysical procedure was used. In each trial a body stimulus appeared on the screen for 1 s, followed by a gray screen and a brief tone to indicate that a response was required. Participants used a two-button mouse to indicate whether they thought the image seemed larger or smaller than the unmanipulated images to which they had been familiarized. Participants were encouraged to respond as quickly and as accurately as possible, following which the next trial began after a 100 ms inter trial interval.

The level of stimulus expansion/contraction of the test stimulus used in each trial was guided by a double interleaved 1-up-1-down adaptive staircase routine. Hence, if a participant rated an image as appearing expanded (contracted), the next time that body was seen a more contracted (expanded) version was presented and vice versa. This continued until a “reversal” was obtained at which point a more expanded (contracted) version was presented. Beginning with a ±50% adjustment in expansion/contraction (e.g., going from −30% contraction to +20% expansion), the step size was reduced by 15% after each reversal until the minimum 5% step size was reached (after the third reversal). Each staircase terminated after eight reversals at which point the mean distortion level of each participant's last six reversals was calculated to represent the PSN. Each staircase proceeded until it reached a maximum of 30 trials. If participants did not reach eight reversals, with consistent responses to the extreme test stimulus, the PSN was recorded at the appropriate extreme of the range of test stimulus values. Two interleaves were run concurrently in a randomized fashion for each test body with one starting at −30 and one at +30%, with each participant's baseline PSN calculated as the average of the PSNs from each staircase.

Adaptation data collection followed the baseline block. This progressed in the same manner as baseline testing with the following exceptions. Testing began with a 120 s initial adaptation phase, during which participants were exposed to alternating images of self and other (2 s each presentation: 60 s total for each stimulus type). In between each trial, 6 s of top up exposure, with the two alternating adaptation stimuli visible for 1 s each (total 3 s), ensured that adaptation was maintained before the next test body was presented.

### Results and discussion

**Figure 3:**
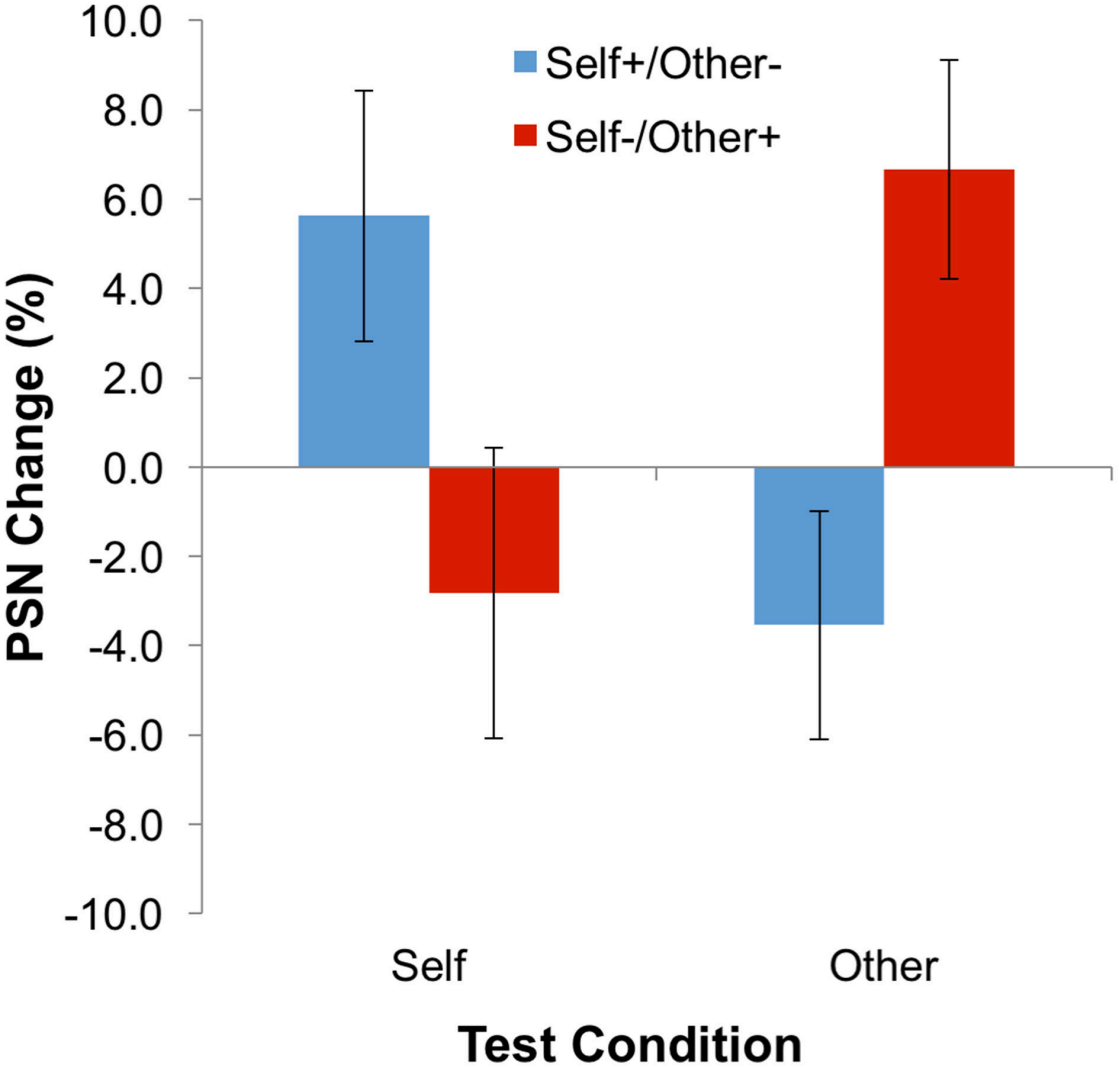
**Results for Experiment 1: Contingent Adaptation**. Error bars represent ±1 SEM.

The main dependent variable—the change of PSN between baseline and adaptation blocks—is plotted in Figure 3. Means, calculated across participants, are all positive following adaptation to expanded stimuli (i.e., the “self” test condition for the “self-expanded/other-contracted” adaptation group, and the “other” test condition for the “self-expanded/other-contracted” adaptation group). Hence, unmanipulated bodies appeared to be abnormally thin, and therefore needed to be expanded to appear normal. The opposite pattern of results is observed following adaptation to contracted stimuli (the “other” test stimulus for the “self-expanded/other-contracted” adaptation group, and the “self” test stimulus for the “self-expanded/other-contracted” adaptation group). These informal observations were confirmed by a statistically significant interaction between adaptation condition and test stimulus in a 2-way ANOVA [*F*_(1, 22)_ = 10.19; *p* = 0.004; $\eta_{\text{p}}^{2}$ = 0.317], demonstrating the presence of a contingent aftereffect. As expected, neither the main effect of adaptation condition [*F*_(1, 22)_ = 0.003; *p* = 0.958; $\eta_{\text{p}}^{2}$ < 0.0005] nor of test stimulus [*F*_(1, 22)_ = 0.113; *p* = 0.740; $\eta_{\text{p}}^{2}$ = 0.005] proved to be significant.

Although the size of the shifts in PSN were small (compare with examples of stimuli given in Figure 1), the significant effects for these data demonstrate, for the first time, the presence of simultaneous aftereffects in opposite directions contingent on stimulus identity. This supports the notion that the perception of body size is mediated, at least to some extent, by dissociable neural mechanisms that can be adapted independently of each other. In addition, this is consistent with the idea that different bodies are compared to different perceptual norms.

## Experiment 2: Cross adaptation

The observation that contingent body size aftereffects can be established suggests that the neural processes underlying the perception of body size for self and other are at least to some degree separate. However, the degree of separation is not clear. While these results are consistent with a system in which the processing of bodies of self and other are mediated by completely independent processes, it is also possible that they represent the operation of a system with overlapping mechanisms. For example, if perceived body size is determined by neural structures that are strictly selective for self and other in concert with others that are not selective for stimulus type, only the latter pool of neurons would experience any “cancelation” of the opposite adaptation effects. This would lead to a small net aftereffect demonstrating the effects in stimulus selective neurons. We sought to further investigate the degree of independence of the mechanisms underlying the processing of these stimulus categories using a cross adaptation paradigm, using a single adapting stimulus, and assessing its effects on both stimulus types.

If the same neural structures are involved when judging the size of the self and other, then aftereffects of the same magnitude would be expected regardless of whether the same stimulus is used for adaptation and test (e.g., adapt with self, test with self; or adapt with other, test with other), or whether these two stimuli are dissimilar (e.g., adapt with self, test with other; or adapt with other, test with self). This is known as cross adaptation. However, if the neural mechanisms for the perception of body size in self and other are entirely independent, then aftereffects established with one adaptation stimulus should be seen when testing with the same stimulus type only, with no transfer of this aftereffect to a test stimulus of a different type. The observation of partial transfer, i.e., significant, but nevertheless smaller aftereffects when adapting and testing with different stimulus types, would indicate an intermediate degree of overlap between the neural populations.

Although previous experimental reports of body size aftereffects evince a causal link between exposure and the perception of body shape and size, for this aftereffect to be considered a realistic model of real-world cases of body image distortion, it would be necessary to demonstrate transfer between the identity of other, typically unfamiliar bodies (e.g., models in fashion magazines) and images of one's self. In addition to reinforcing the link between laboratory results and real-world body image distortion, such a cross adaptation effect would allow us to infer a degree of overlap between the neural substrates that are involved in processing each stimulus type that could not be revealed by the contingent adaptation paradigm. Although one study (Hummel et al., 2012b) has assessed the degree of transfer between identities, this study used grayscale images of the self that did not show any explicit identifying information (torso only), and which the participants may not have perceived as representing their own body. Furthermore, simple adaptation and cross adaptation experiments were performed under different conditions (e.g., substantial differences between the clothing worn by bodies serving as adaptation stimuli) in Hummel and colleagues' research, rendering comparisons problematic and making inferences regarding common/independent mechanisms equivocal. In the following experiment, we use easily identifiable color photographs of the full body including the photographic subject's head and face, captured under identical conditions, to compare the magnitude of simple and cross adaptation aftereffects.

### Methods

Methodological details for this experiment were identical to those for Experiment 1 except in the following respects. We used a 2 × 2 × 2 mixed factorial design with one within-subjects and two between-subjects independent variables. The within-subjects variable—test identity—involved two levels: “self” and “other.” The first between-subjects variable—adaptation identity—had the same two levels: “self” and “other,” while the second between-subjects variable—adaptation direction—also had two levels: “expanded” and “contracted.” Thirty-five female participants were randomly allocated to each of these four conditions. Initial adaptation lasted 120 s, during which participants viewed a single adaptation stimulus featuring a particular combination of adaptation direction (expansion or contraction) and adaptation identity (self or other). In between each trial, 6 s of top up exposure ensured that adaptation was maintained before the next test body was presented.

As in experiment 1, we expect to see a main effect of adaptation direction, as adaptation to an expanded and a contracted image are expected to yield opposite effects. While complete transfer of the aftereffect between others and the self would appear unlikely given the results of experiment 1, it would be revealed by equivalent adaptation effects for both test stimuli regardless of which adaptation stimulus is used, and hence no additional significant ANOVA effects. However, partial transfer (i.e., observations that aftereffects are less intense when adapting and testing with different stimuli, compared to adapting and testing with the same stimuli) would be represented by a crossover interaction between adaptation identity and test identity for the expanded adaptation conditions, with the mirror-image pattern of results for contracted adaptation conditions. This corresponds to a three-way interaction between the three independent variables.

### Results and discussion

**Figure 4:**
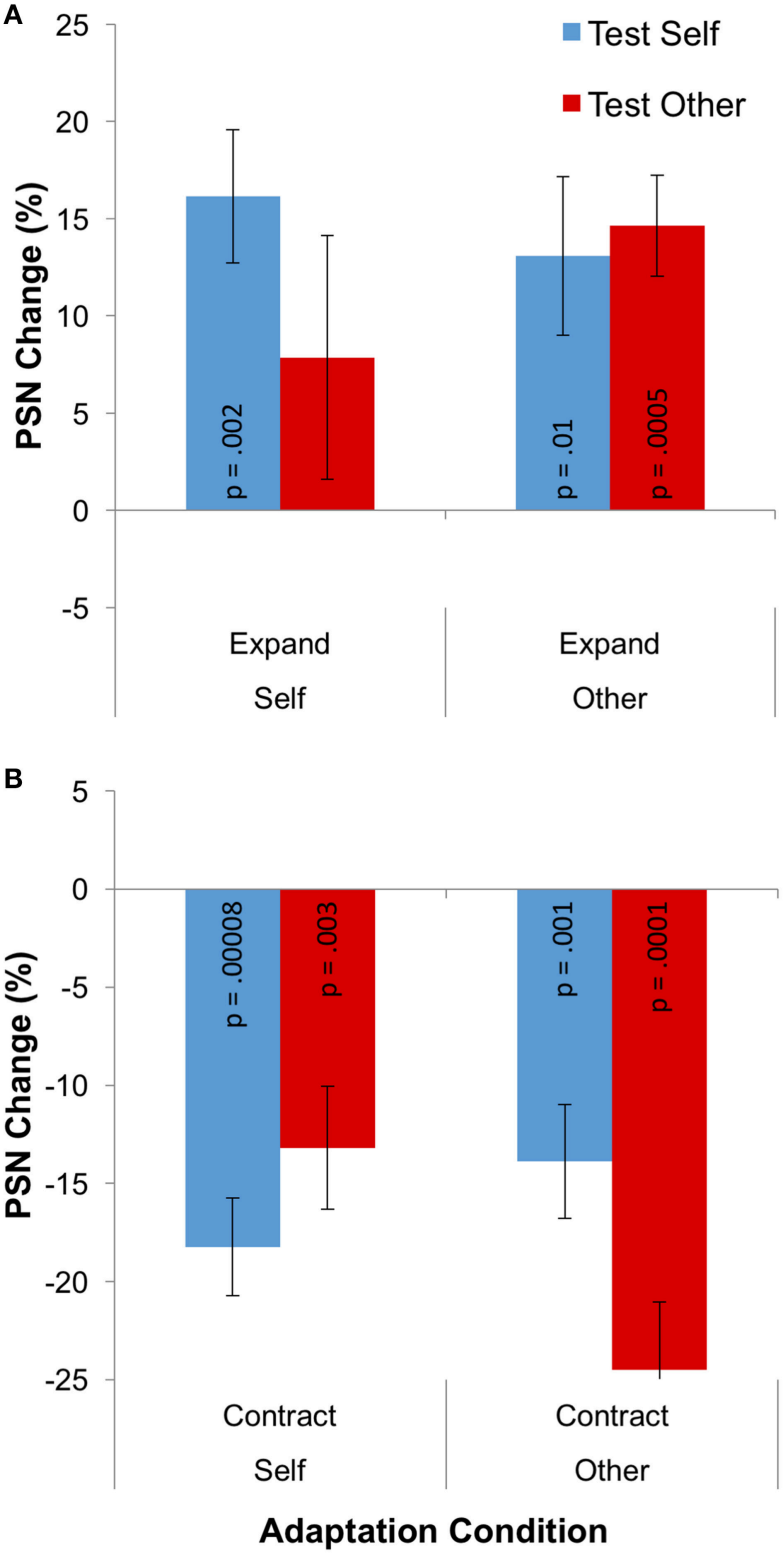
**Results for Experiment 2: cross adaptation**. **(A)** PSN change for expanded adaptation conditions. **(B)** PSN change for contracted adaptation conditions. The *p*-value for each individual one-sample *t*-test is given, to one significant figure (without correction for multiple comparisons), on each data column. Error bars represent ±1 SEM.

The change of PSN from baseline that is caused by adaptation is plotted in separately for the different adaptation directions in Figure 4. Means, calculated across participants, are all positive following adaptation to expanded stimuli, indicating that unmanipulated stimuli appeared to be abnormally thin, and therefore needed to be expanded to appear normal (Figure 4A). The opposite pattern of results is shown following adaptation to contracted stimuli (Figure 4B). In addition, aftereffects appear to be smaller when different identities are used as adaptation and test stimuli. These observations were confirmed in a 2 × 2 × 2 mixed ANOVA, which showed a significant main effect of adaptation direction [*F*_(1, 31)_ = 94.498; *p* < 0.0005; $\eta_{\text{p}}^{2}$ = 0.753] and a significant three-way interaction between adaptation direction, adaptation identity and test identity [*F*_(1, 31)_ = 11.731; *p* = 0.002; $\eta_{\text{p}}^{2}$ = 0.275].

**Figure 5:**
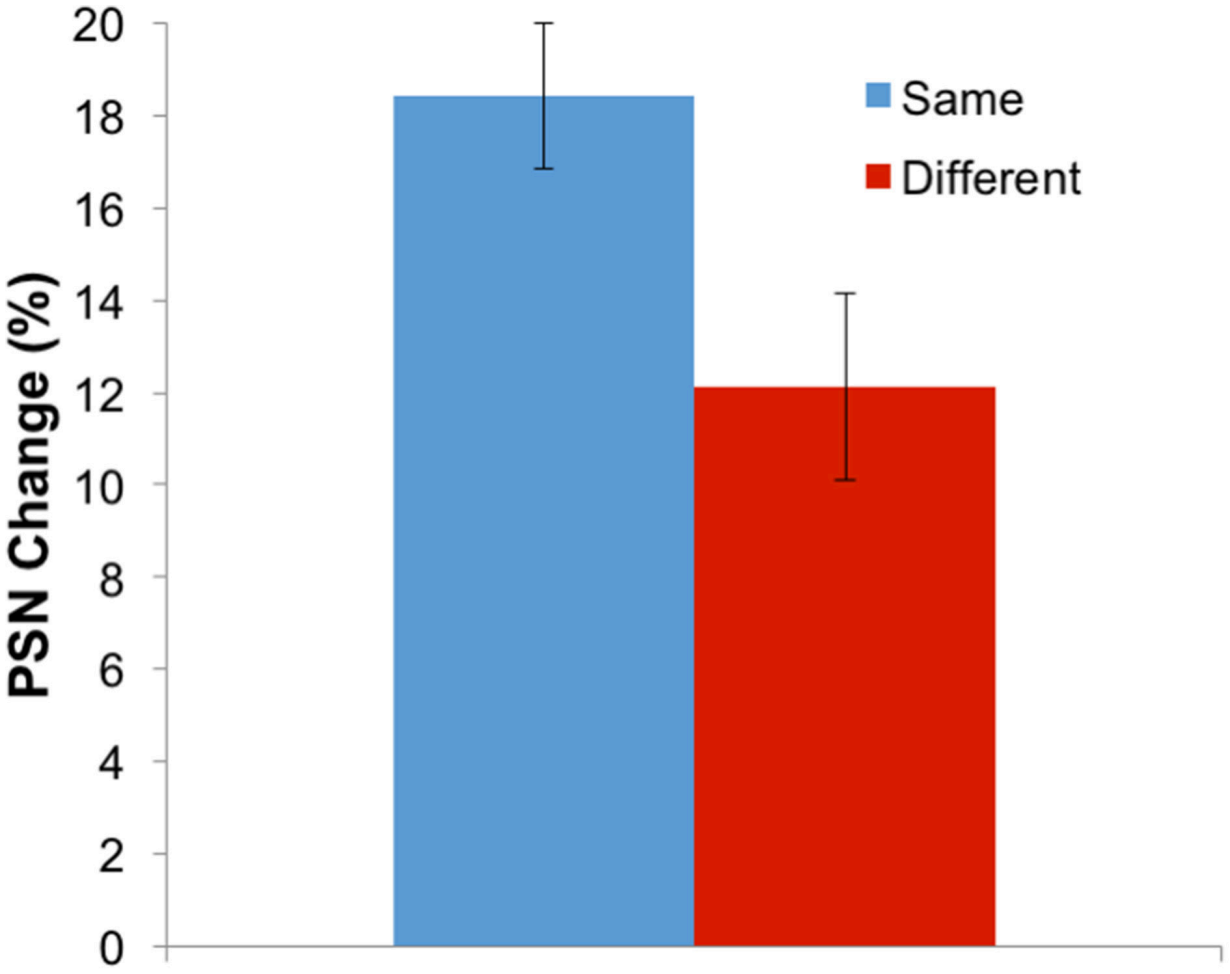
**Results for Experiment 2 combined and replotted**. Aftereffect magnitude (in the expected direction) for adaptation and test either with the same or with different stimulus identities. Error bars represent ±1 SEM.

While the three-way interaction demonstrates that aftereffects have a different magnitude when adapting and test stimuli involve the same body identity (self or other), this can be more clearly communicated by replotting the data. Given that the different adaptation directions (expanded and contracted) are expected to produce different directions of aftereffect, these conditions can be combined by multiplying all PSNs from the expanded adaptation conditions by −1. Further, the remaining four conditions involving the use of self or other as adaptation and test stimuli can be combined into two conditions which involve adapting and testing with the same, or with different body identities. Such a plot is shown in Figure 5, demonstrating that the change in PSN is smaller when adapting and testing with different stimuli, such that the transfer of the aftereffect is only partial, at 65%. A paired-samples *t*-test confirms the significance of this finding [*t*_(34)_ = 3.378, *p* = 0.002].

While the effects detailed above are relevant to the issue of the independence of neural mechanisms, to test the viability of visual adaptation as a model for various types of real-world body image distortion, we wished to assess the presence of significant aftereffects in each individual condition. For this reason, 8 one-sample *t*-tests were performed. These yielded significant results in all but one instance, namely the condition in which participants viewed an expanded image of themselves, but were tested with an image of an other's body (*p* = 0.249). All other conditions produced *p*-values in the range 0.00008–0.012, as shown in Figure 4.

Overall, these results confirm that adaptation to images that have been manipulated to appear thinner or fatter than normal are effective in creating aftereffects of perceived body size. Further, effects caused by prolonged exposure to distorted images of the self can transfer to the bodies of others and, more importantly, exposure to thin or fat unfamiliar others can transfer to the self. The latter observation may have implications for the occurrence of body image distortion in real-world settings, including clinical psychology settings.

## General discussion

In two experiments, we have demonstrated the phenomenon of body image misperception through exposure to bodies that had been digitally manipulated to appear fat or thin. The aftereffect of exposure to such images for a period as short as 1 min was sufficient to cause subsequently seen images to have a contrasting appearance, such that exposure to thin images made unmanipulated images appear enlarged, and vice versa. While effects such as these are not unprecedented (Winkler and Rhodes, 2005; Glauert et al., 2009; Hummel et al., 2012a,b, 2013; Robinson and Kirkham, 2014; Brooks et al., in press; Mohr et al., 2016), experiment 1 is the first to show simultaneous aftereffects of exposure to fat and thin images contingent on the identity (self vs. other) of the test stimulus. This adaptation effect was successful regardless of whether images of the self were thin and images of the other identity were fat or vice versa. This suggests that the two identity types are compared, at least to some extent, against different perceptual/psychological norms, while establishing at least a partial dissociation of the neural mechanisms encoding body size for self and other. Extending this finding, experiment 2 investigated the extent to which the mechanisms encoding the body size of self and other are separate. The results demonstrate substantial transfer of the aftereffect between images that explicitly depict the participant's own body and those of other bodies (see also Hummel et al., 2012b). This cross adaptation effect is reciprocal: the effect transfers from exposure to others' bodies affecting the perception of one's own body, and from exposure to one's own body influencing the perception of another's body. However, since the effect was smaller when the body used for exposure and the body used for testing were different, compared to when the same stimulus is used for both, the transfer is not complete. That the aftereffect showed only partial transfer (in this case, a 65% transfer) implies that the neural mechanisms responsible for encoding body size for one's own body and the body of others show a degree of overlap.

While the current study presents evidence of dissociable neural populations processing the size of one's own and others' bodies, it is not able to locate the relevant structures. Previous studies have identified various cortical areas that are particularly active when bodies are viewed (compared to their responses to other stimuli). These areas include the extrastriate body area (EBA), the fusiform body area (FBA), the right middle occipital gyrus (rMOG), the lateral occipital cortex (LOC), the middle frontal gyrus (MFG) and the inferior parietal lobe (IPL), amongst others. Some of these areas [EBA and to some extent FBA: (Hodzic et al., 2009; Aleong and Paus, 2010); LOC and MFG: (Mohr et al., 2011)] appear to show a modulation of activity when viewing bodies of different sizes, making them candidates for the mediation of body size perception. Other areas (right IPL, right EBA, left FBA: Hodzic et al., 2009) show selectivity for different identities. Further, changes in the neural responses that accompany aftereffects of perceived body size have been demonstrated by Hummel et al. (2013). These authors found reductions in BOLD responses in the FBA and the rMOG with repeated presentation of images of their own bodies, while EBA showed no such adaptation effect. However, as this experiment lacked a condition wherein bodies were presented but no size aftereffect would be expected (e.g., using a normal body as the adaptation stimulus), it is not clear whether the imaging results reflect body size judgments, or simply the expected general reduction in activity that accompanies stimulus repetition. Aleong and Paus (2010) also demonstrated adaptation to body stimuli depicting other unfamiliar individuals in areas EBA and FBA, although the relationship between this activity and size aftereffects is similarly unclear. Importantly, these authors also demonstrated a partial release from this adaptation effect when the subject's identity was changed. The latter finding indicates some sensitivity of EBA and FBA to this attribute of bodies, and raises the possibility that this activity underlies the separate processing of the body size of self and other identified in the current study.

While the current research employed many different identities, each participant saw only two: their own body and the body of an unfamiliar other. This leaves open the possibility that the effects seen here are not specific to the self, but rather would be observed regardless of the two specific identities used, so long as these are unambiguously distinct. It is noteworthy that similar caveats have been acknowledged for a related study involving the perception of the faces of the self and other (Rooney et al., 2012). While further research replacing the body of the self with the body of a second unfamiliar individual would address this question, we chose to concentrate on the examples of aftereffect transfer that may be most relevant to real-world examples of body image misperception in the current study. The observation that adaptation to thin others causes observers to regard their own objective bodies as larger than normal corresponds to the established, real-world phenomenon whereby some individuals—including those with eating disorders and other clinical manifestations of body image disturbance—tend to over-estimate their body size (Groesz et al., 2001; Levine and Murnen, 2009). In this case, where exposure to the thin ideal in the mainstream media takes the place of our 2-min adaptation period, and self-observation in a mirror takes the place of our on-screen test stimuli. We have also presented evidence that equal and opposite effects can be seen when the exposure is to larger individuals, such that one's own normal body appears abnormally thin. This size underestimation of body size may correspond to effects reported for overweight adults residing and/or working in areas with high obesity prevalence (Burke et al., 2010; Powell et al., 2010), and perhaps children and adolescents with overweight friends and/or parents (Maximova et al., 2008). While experimental tests of these effects have been conducted (e.g., Robinson and Kirkham, 2014), to our knowledge the current study is the first to demonstrate the transfer of the aftereffect between exposure to the large bodies of others and judgments of one's own body.

While the duration of exposure to adapting images in the present study was relatively brief, yet sustained (e.g., 1 min of constant stimulation), it is clear that patterns of exposure for images in daily life are rather different. Media saturation may lead to many years of exposure to “ideal” bodies, although exposure is unlikely to be constant during this period. However, these differences do not constitute a serious challenge to the viability of visual adaptation as an experimental model of body image distortion. Although we chose a time course that suited our experimental investigation, in general, adaptation effects have been demonstrated across many different timescales, involving exposure and aftereffect durations ranging from milliseconds to months (Webster, 2011, 2015). In general, longer durations of adaptation are associated with longer lasting aftereffects, while frequent re-exposure has been identified as a factor that tends to further prolong the duration (Ditye, 2015). Hence, it is reasonable to hypothesize that “real world” body adaptation effects shown by some individuals may be larger than those induced in the current research.

A potential limitation involves our participant sample, who were all young women taken from an undergraduate sample. In part, this sub-group may be the most relevant, as they are considered to be at higher risk of developing body dissatisfaction issues, eating disorders and associated pathologies. This was deemed appropriate for an initial study, given that body size misperception and associated pathologies are overrepresented in this demographic (Mond et al., 2013; Mitchison and Mond, 2015). Further, there are specific reasons for the exclusion of certain other groups. Older participants were excluded in view of the fact that body shape changes with age, as does the way in which bodies change as they increase in mass. Although the effect of adaptation to self and other has not yet been tested in males, other data from related studies in our laboratory show that young males are also susceptible to body size aftereffects (Brooks et al., in press; Stephen et al., in submission). While we have no reason to believe that the results reported here would be any different either for older female observers or for males, further research will be needed to test this hypothesis.

The fact that participants with high levels of body image concern were not specifically targeted in the current study also needs to be considered when interpreting the current study findings. It has recently been reported that, despite success for normal observers, experimenters were not able to adapt eating disorder patients to images of thin bodies to create an aftereffect that their own body is larger (Mohr et al., 2016). While there was variance in results within this group, there was also a significant negative correlation between aftereffect size and scores on eating disorder questionnaires. However, the same group of participants did show aftereffects in the opposite direction when exposed to fat stimuli. As the authors of this study noted, these findings are consistent with the idea that those with clinical issues may already be “pre-adapted” through their usual daily diet of body images, precluding further adaptation effects.

While there are substantial differences between the strict laboratory conditions that were imposed for this experiment and real-world examples of body image distortion, and although our participants were clearly very different from those who suffer from clinical forms of body image disturbance, we believe that this paradigm may have utility in terms of the development of an experimental model of body image distortion. With further experimentation to confirm the viability of the paradigm, it may be possible to make observations that can inform health promotion programs and/or clinical practice. As a preliminary example, if exposure to extreme body types can contribute to body image misperception, it may be considered inadvisable to assemble sufferers with the same extreme body types, such as those with the eating disorder anorexia nervosa, for group treatment. Although some authors have already voiced concerns over this relatively common practice, objections usually relate to examples of weight loss competition, increased social pressure from within the inpatient group, or to collaboration over weight loss strategies between sufferers of anorexia nervosa (Colton and Pistrang, 2004; Vandereycken, 2011; Allison et al., 2014). The results of this study suggest that such congregations could also be harmful in the sense of exacerbating individuals' body image disturbance, further reducing motivation to gain weight. While visual adaptation may be substantial under such circumstances, its effects are unlikely to be elucidated through the use of self-report questionnaires, diagnostic interviews or other methods that are commonly used in these situations.

## Author contributions

KB: responsible for the conception and design; the acquisition, analysis and interpretation of data; original drafting and final approval of the submitted version. JM: substantial contributions to the interpretation of data, critical revisions for important intellectual content, and final approval of the submitted version. RS: substantial contributions to the interpretation of data, critical revisions for important intellectual content, and final approval of the submitted version. IS: substantial contributions to the interpretation of data, critical revisions for important intellectual content, and final approval of the submitted version.

### Conflict of interest statement

The authors declare that the research was conducted in the absence of any commercial or financial relationships that could be construed as a potential conflict of interest.
